# Photodynamic nasal SARS-CoV-2 decolonization shortens infectivity and influences specific T-Cell responses

**DOI:** 10.3389/fcimb.2023.1110467

**Published:** 2023-01-25

**Authors:** Alejandro Fernandez-Montero, Jon Zuaznabar, Manuel Pina-Sanchez, Sheila Maestro, Loreto Martin-Navarro, Natalia Muñoz-Rodríguez, Cristina Olagüe, Marta Pastrana, Maria Martínez-Fernández, Gracian Camps, Jose Antonio Rodriguez, Francesco P. Marchese, Jon Zazpe, Marta Pozuelo, José Luis Del Pozo, Jorge Quiroga, Antonio Pineda-Lucena, Gabriel Reina, Jack Kolenda, Laura Moreno-Galarraga, Gloria Gonzalez-Aseguinolaza, Marta Rua, Cristian Smerdou, Francisco Carmona-Torre, Josepmaria Argemi

**Affiliations:** ^1^ Department of Occupational Medicine, University of Navarra, Pamplona, Spain; ^2^ COVID19 Unit, Clinica Universidad de Navarra, Pamplona, Spain; ^3^ Instituto de Investigación Sanitaria de Navarra (IdisNA), Pamplona, Spain; ^4^ Internal Medicine Department, Clinica Universidad de Navarra, Pamplona, Spain; ^5^ Microbiology Department, Clinica Universidad de Navarra, Pamplona, Spain; ^6^ Division of Gene Therapy and Regulation of Gene Expression, CIMA, Universidad de Navarra, Pamplona, Spain; ^7^ Laboratory of Atherothrombosis, Program of Cardiovascular Diseases, CIMA Universidad de Navarra, Pamplona, Spain; ^8^ Centro de Investigacion Biomedica en Red (CIBER-CV), Madrid, Spain; ^9^ Genomics Unit, CIMA Universidad de Navarra, Pamplona, Spain; ^10^ Bioinformatics Platform, CIMA Universidad de Navarra, Pamplona, Spain; ^11^ Infectious Diseases Division, Clínica Universidad de Navarra, Pamplona, Spain; ^12^ Molecular Therapeutics Program, CIMA Universidad de Navarra, Pamplona, Spain; ^13^ Department of Otolaryngology Head and Neck Surgery, University of Toronto, Toronto, ON, Canada; ^14^ Department of Pediatrics, Complejo Hospitalario de Navarra, Servicio Navarro de Salud, Pamplona, Spain; ^15^ Liver Unit, Hepatology Program, Clinica Universidad de Navarra, CIMA Universidad de Navarra, Pamplona, Spain

**Keywords:** COVID19, SARS-CoV-2, photodynamic therapy, photodisinfection, infectivity, anti-spike antibody, inmunity

## Abstract

**Background:**

The main objective was to evaluate the efficacy of intranasal photodynamic therapy (PDT) in SARS-CoV-2 mildly symptomatic carriers on decreasing the infectivity period. SARS-CoV-2-specific immune-stimulating effects and safety were also analysed.

**Methods:**

We performed a randomized, placebo-controlled, clinical trial in a tertiary hospital (NCT05184205). Patients with a positive SARS-CoV-2 PCR in the last 48 hours were recruited and aleatorily assigned to PDT or placebo. Patients with pneumonia were excluded. Participants and investigators were masked to group assignment. The primary outcome was the reduction in *in vitro* infectivity of nasopharyngeal samples at days 3 and 7. Additional outcomes included safety assessment and quantification of humoral and T-cell immune-responses.

**Findings:**

Patients were recruited between December 2021 and February 2022. Most were previously healthy adults vaccinated against COVID-19 and most carried Omicron variant. 38 patients were assigned to placebo and 37 to PDT. Intranasal PDT reduced infectivity at day 3 post-treatment when compared to placebo with a β-coefficient of -812.2 (CI95%= -478660 – -1.3, p<0.05) infectivity arbitrary units. The probability of becoming PCR negative (ct>34) at day 7 was higher on the PDT-group, with an OR of 0.15 (CI95%=0.04-0.58). There was a decay in anti-Spike titre and specific SARS-CoV-2 T cell immunity in the placebo group 10 and 20 weeks after infection, but not in the PDT-group. No serious adverse events were reported.

**Interpretation:**

Intranasal-PDT is safe in pauci-symptomatic COVID-19 patients, it reduces SARS-CoV-2 infectivity and decelerates the decline SARS-CoV-2 specific immune-responses.

## Introduction

The COVID-19 pandemic has affected humans of all ages and conditions across the globe (Hu et al., 2021). Thanks to the rapid development of vaccines, a broad population immunity, at least against lethal variants, has been achieved in many countries (Watson et al., 2022). Nevertheless, even the most efficacious mRNA vaccines, despite achieving a significant reduction in morbidity and mortality, are failing to prevent new pandemic waves and it is uncertain what the level of protection will be against future viral genotypes (Andrews et al., 2022; Kirsebom et al., 2022).

Non-Pharmaceutical Interventions (NPI) including face masks, social distancing, individual isolation or lockdowns have shown to be effective in reducing the reproduction number (R) of SARS-CoV-2 (Li et al., 2021). Although lack of compliance with NPI in the first waves of the pandemic has been associated with significant production loss (Ohrnberger et al., 2021), the real-life impact of loss of active workforce in non-essential jobs is uncertain therefore, treatments to reduce viral colonization in upper airways have also been proposed as complementary methods. RNA sequencing studies showed a particularly high expression of viral entry factors, with the most affected target being the angiotensin converting enzyme 2 (ACE2) receptor located in the nasal epithelium (Vieira Braga et al., 2019; Deprez et al., 2020; Sungnak et al., 2020). While these data were initially useful to understand both the rapid spread and predominantly pneumonic phenotype of COVID-19, they also underlined the importance of the nose as the entry site and potential primary replication reservoir.

Intranasal photodynamic therapy (PDT) is an antimicrobial strategy based on the activation of a photoactive methylene blue-based compound within the anterior nares by a cold laser light (Zolfaghari et al., 2009). Nasal decolonization of *Staphylococcus aureus via* PDT has been shown to be a safe and effective strategy to minimize the risk of surgical site infections (Bryce et al., 2014). Recent reports suggest important anti-SARS-CoV-2 activity in *ex vivo* models (Lobo et al., 2022) and extensive *in vitro* PDT testing against wild strains of coronaviruses has been recently published (Arentz and von der Heide, 2022). Very recently, a proof-of-concept feasibility and safety study in humans within different hospital settings has been conducted showing signals of decay in infectivity assays (Pires et al., 2022).

We hypothesized that an early eradication of viral particles from the anterior nares by nasal-PDT could reduce viral spread and infectivity in SARS-CoV-2 patients and that photochemical internalization (PCI) of post-treatment residual antigens into dendritic cells might impact on patients’ immune responses.

## Methods

### Study design

This study consisted of a single centre, randomized, placebo-controlled, single-blind, clinical trial of methylene blue-based PDT in SARS-CoV-2 positive individuals. We aimed to evaluate safety, efficacy, and antiviral immune responses.

Every participant was informed about the rationale and the characteristics of the study protocol and gave informed consent. The study, designed to follow CONSORT Statement criteria (http://www.consort-statement.org/), was carried out at the Clinica Universidad de Navarra, a tertiary teaching hospital in Pamplona, Spain, in accordance to the principles expressed in the Declaration of Helsinki. The study protocol was approved by the local Human Research Ethics Committee (registry number: EC_2021/1) and registered in ClinicalTrials.gov (NCT05184205, “SARS-PDT01”).

### Patients

Participants were recruited from the University of Navarra COVID19 Program (Gil-Conesa et al., 2022) and the *Clínica Universidad de Navarra* surveillance program. Both programs were established in 2020 to perform active surveillance with the intention of avoiding viral outbreaks within the University campus and the hospital (Gil-Conesa et al., 2022).

Inclusion criteria included to be over 18 years-old and to have a positive SARS-CoV-2 PCR (<27 cycles) in the last 48 hours. Concomitant medications except angiotensin receptor blockers or immunosuppressant agents were allowed. All patients consented to receive either PDT or placebo equivalent. Patients with severe comorbidities or moderate-severe COVID-19 were not included. Individuals who reported inability to tolerate insertion of the light illuminator due to oronasal size, shape, or anatomical variants, those with known allergic reactions to components of the nasal decolonization treatment including methylene blue, and those unable to attend the follow-up appointments were excluded.

### Randomisation, controlling and masking

Participants were randomly assigned to PDT or placebo group (1:1). The investigator who performed randomization did not apply treatments nor collected biological samples. Participants and investigators assessing outcomes were masked to group assignment.

The control treatment was performed using saline solution and a sham treatment with a switched-off laser illuminator device, with the patient blinded to the intervention by polarizing glasses. Placebo-treated participants followed the same timing and cycle protocol as the PDT- group.

### Procedures

Photodynamic treatment was performed using the CE-marked Steriwave™ Nasal Photodisinfection System (NPS, SW4000, Ondine Biomedical Inc, Vancouver, BC, Canada). This is a Class II Medical Device that includes a power source, a Nasal Light Illuminator (NLI) and a methylene blue formulation (MBF) approved in Canada and Europe for the decolonization of the anterior nasal passages. The topically applied MBF binds microbial components, and the illuminating red light is absorbed by the photosensitizer molecules, producing reactive oxygen species responsible for the lethal microbial disruption. Selectivity is produced by electrostatic differentiation of the cationic photosensitizer to microbes versus generally zwitterionic host tissues.

Nasopharyngeal (NP) swabs were performed before treatment and on days 3 and 7. NP specimens were collected in Universal Transport Media (Vircell) and used for RNA extraction (TANBead^®^ Nucleic Acid Extraction Kit) and RT-PCR (Cepheid Xpert Xpress SARS-CoV-2, genes E and N) and were kept in UTM for *in vitro* infectivity assays in Biosafety Level 3 facilities. An aliquot extracted from baseline NP swabs was used for SARS-CoV-2 sequencing. All procedures were performed by trained healthcare professionals.

The procedure was initiated by swabbing the MBF inside the patient’s anterior nares, including nostrils and nasal passages. The dual-channel NLI was connected to the power source and inserted into the patient’s nostrils. A 4-minute illumination cycle was applied three times using new MBF after each cycle. This 4 min x 3 scheme was repeated on day 2 and 3. In total, each patient was treated for 36 min. NLI channels were labelled with “L” and “R” for the left/right nostril and a a new set of NLI was used each day to avoid self-contamination.

### Outcomes

The primary study outcome was infectivity reduction after 3 days of treatment. Because prior works had demonstrated variable PCR response to viral killing due to genomic remnants still permitting primer binding (Bullard et al., 2020; La Scola et al., 2020; Walsh et al., 2020), *in vitro* infectivity assays with Vero-E6 cells were preferred to RT-PCR as an endpoint, although both techniques were performed in each sample. Secondary outcomes included safety and infectivity reduction at other time points (7 and 14 days). Biological correlates included analysis of immunogenicity against Nucleocapsid, Spike and total SARS-CoV-2 genome at 10 and 20 weeks and the genomic sequencing of the specimens.

### Clinical follow-up

Patients were asked about COVID-19 symptoms on the baseline visit, and at day 3 and 7. The list of symptoms was based on previous studies (Lan et al., 2020) included sore throat, chills, cough, dyspnoea, chest tightness, temperature, fatigue, muscle pain, loss of smell, loss of taste, headache, gastrointestinal symptoms, difficulty sleeping, general malaise and nasal congestion. Symptoms were graded on a four-category scale (absent, mild, moderate, or severe).

### Safety analysis

To evaluate safety, immediate and delayed local effects were collected and evaluated. The severity of these and the likelihood of being treatment-related were also noted. The safety questions were asked immediately after treatment and at each subsequent visit.

### Covariates

Covariates collected included gender, age, COVID-19 vaccination and booster doses, previous Covid-19 infection, weight, height and vital signs such as body temperature, heart rate, blood pressure and blood oxygen saturation.

### Statistical analysis

The sample size calculation was performed based on differences found in the pre- and post-intervention delta-Ct of a preliminary *ex vivo* study. At 99% power only a small sample size was needed to demonstrate microbiological efficacy (6 in each group). It was assumed that subjects would present with a wide range of ages and comorbidities. We also expected a spontaneous decline in viral load in our study population (since they consisted of mostly vaccinated healthy individuals). In addition, a 10% dropout rate was considered; we therefore proposed a sample size of 100 patients. Due to the rapid fade of the sixth COVID-19 wave in Spain (December 2021-February 2022) we were unable to recruit the full sample size; 78 patients were randomized, and 75 patients completed the study.

To compare quantitative variables Mann-Whitney U test was performed for those that did not follow normality and Student t test for those that did. Wilcoxon test was performed when paired samples were compared. Medians and interquartile ranges and mean and standard deviations were calculated, respectively. Chi^2^ test was performed for qualitative variables. In the infectivity trial analyses, multiple linear regression model was performed, and Beta coefficients and their respective 95% confidence intervals (95% CI) were calculated. To assess the ability to reduce contagion, multi-adjusted logistic regression models were performed, and Odds Ratios (OR) and 95% CI were estimated. Both models were adjusted for gender, age, initial symptoms, COVID-19 vaccination status and previous SARS-CoV-2 infection. All p values presented are two-tailed. Prism software (GraphPad Software, San Diego, CA) and STATA13.0 were used for statistical analysis.

### *In vitro* infectivity assay

Monolayers of Vero-E6 cells were grown to confluence on 96-well plates and infected with the patients’ samples previously diluted 1:2 with infection medium (Minimum Essential Medium -MEM with 0.2% BSA 0.2%, 2mM glutamine and 20 mM Hepes) and incubated for 4 h at 37°C. After removing the inoculum, Eagle’s MEM with 10% foetal bovine serum and antibiotics was added to each well of infected cells. Non-infected cells were used as negative controls. After 72 h, cells were collected, lysed using Dynabeads™ MyOne™ Silane beads and the number of present SARS-CoV-2 genomes analysed by RT-PCR (Detecting SARS-CoV-2, BGI, genes ORF1ab and Human β–actin). These values are expressed as 2^(-ΔCt)*1000.

### Specific anti-SARS-CoV-2 humoral and cellular responses

To analyse the participant´s serological response, a blood draw was performed 10 and 20 weeks after entering the clinical trial. Anti-SARS-CoV-2 antibody detection was performed using four commercial chemiluminescence tests. First, quantification of total antibodies (IgG+IgM) against the receptor binding domain of SARS-CoV-2 spike (S) using Elecsys^®^ Anti-SARS-CoV-2 S (Roche Diagnostics, Germany). Second, detection of total antibodies (IgG+IgM) against viral nucleocapsid (Anti-N) using Elecsys^®^ Anti-SARS-CoV-2 test (Roche Diagnostics, Germany). Third, Anti-SARS-CoV-2 specific IgG against nucleocapsid and spike proteins using COVID-19 VIRCLIA^®^ IgG Monotest (Vircell SL, Spain). And fourth, Anti-SARS-CoV-2 (IgM+IgA) against nucleocapsid and spike proteins were detected using COVID-19 VIRCLIA^®^ IgM+IgA Monotest (Vircell SL, Spain).

Cell-mediated immune response to SARS-CoV-2 was measured using QuantiFERON^®^ SARS-CoV-2 Starter and Extended Sets (QIAGEN, USA). The Starter Set included specific peptides from the spike antigen (S1, S2, RBD subdomains) to evaluate CD4 (Ag1 tube) and CD4+CD8 (Ag2 tube) T cells immune responses. The Extended Set contained additional specific peptides from the full genome of SARS-CoV-2 (S, N and M domains) to study a complete specific CD4 and CD8 T cell-mediated immune response (Ag3 tube). After inoculation, tubes were incubated and IFN-γ concentrations were measured by QuantiFERON^®^ ELISA (QIAGEN, USA). Samples were considered reactive when any tube showed IFN-γ production above 0.15 IU/mL.

### Sequencing analysis of COVID-19 variants

Libraries were prepared from swab samples using COVIDSeq Assay, (Illumina) and sequenced using a NextSeq2000 (Illumina). Analyses were performed with Kraken (Illumina). The phylogenetic tree was performed with Nextclade.org.

## Results

**Figure 1:**
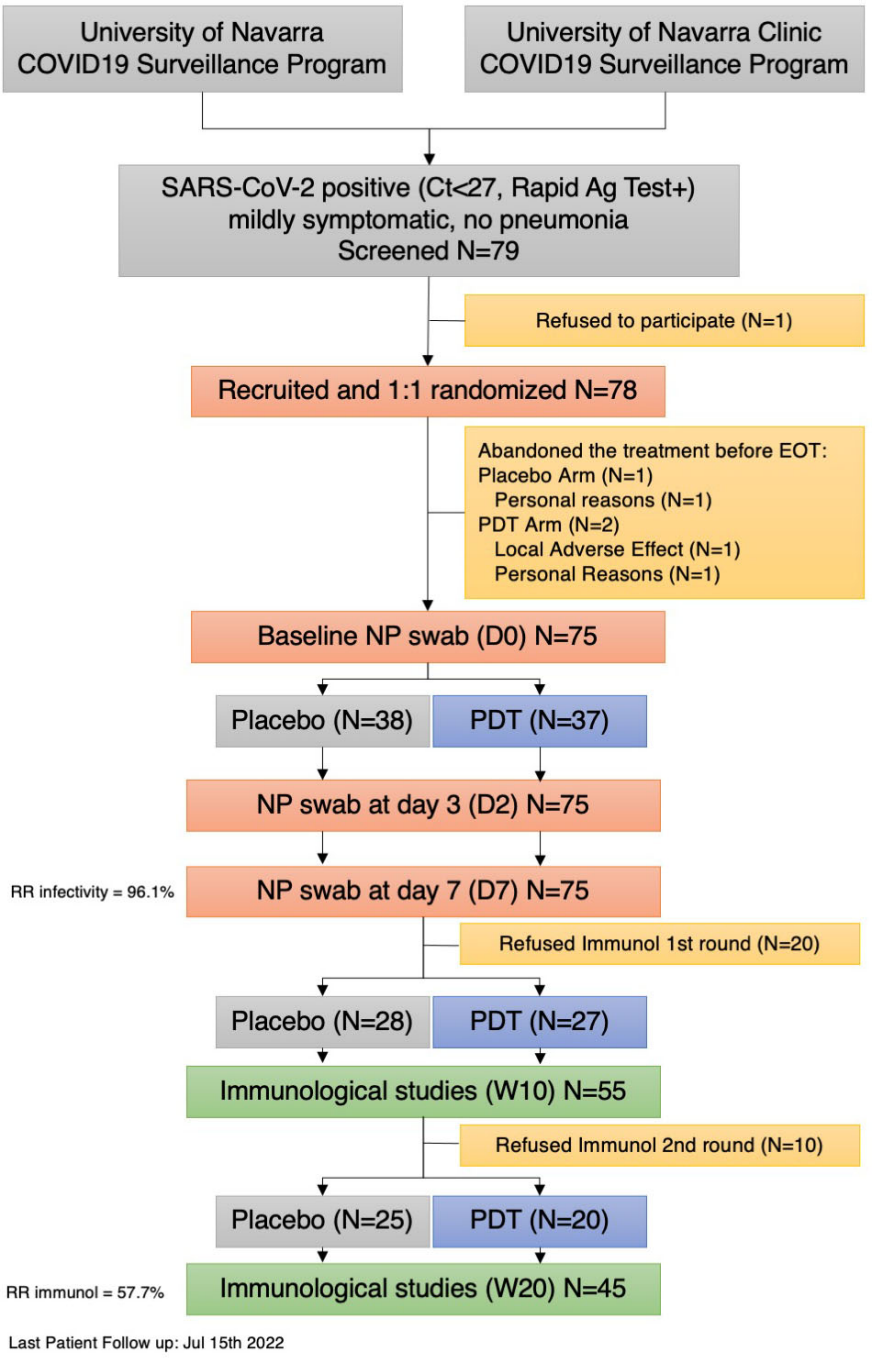
Flow Chart of patient recruitment. RR , Retention Rate Rate; PDT , Photodynamic Treatment; Ag, Antigen; EOT, End of Treatment; D,Day; W, Week.

Seventy-nine patients were screened between December 2021, and February 2022. One was excluded for not meeting the inclusion criteria. Of the 78 patients randomized 1:1, two patients abandoned the treatment group (one for local irritation, the other for personal reasons), and one patient abandoned the placebo group (for personal reasons). Seventy-five patients completed the study and were included in the analyses. Retention rate was 96.1% (**Figure 1**).

The study-population consisted of mostly young healthy adults, with mild symptoms, naïve to natural SARS-CoV-2 infection (79%) with a complete course of vaccination (93%), and a high viral load (**Table 1**).

**Table 1:** Patients’ baseline characteristics.

Characteristic	Placebo	PDT	P
**No.**	38	37	
**Gender (female), No. (%)**	26 (68.4)	24 (64.7)	0.744
**Age, year, median (interquartile range)**	22 (21 – 45)	24 (20 – 43)	0.662
**Smoking, No. (%)**			0.108
**No**	25 (66.8)	32 (86.5)	
**Current**	7 (18.4)	3 (8.1)	
**Former**	6 (15.8)	2 (5.4)	
**BMI, (kg/m^2^), median (interquartile range)**	22.3 (20 – 25)	23.1 (21.6 – 24.4)	0.478
Vulnerability*, No. (%)	6 (15.8)	5(13.5)	0.781
**Received COVID19 vaccine, No. (%)**	35 (92.1)	35 (94.6)	0.666
**Booster doses of COVID19 vaccine, No. (%)**	11 (29)	9 (24.3)	0.651
**Reinfection of SARS-CoV-2, No. (%)**	10 (26.3)	6 (16.2)	0.286
**Number of symptoms, mean (SD)**	5.1 (2.5)	4.2 (2.2)	0.085
**Heart rate, beats/min, mean (SD)**	76.7 (10.7)	77.4 (13.3)	0.812
**SBP, mmHg, median (interquartile range)**	110.5 (105 – 121)	119 (110 – 124)	**0.039**
**DBP. mmHg, median mean (SD)**	72.5 (7)	77.1 (6.2)	**0.004**
**Temperature, C°, median (interquartile range)**	36.2 (35.9 – 36.7)	36.3 (35.8 – 36.6)	0.782
**Oxygen saturation. mean (SD)**	97.9 (1.2)	97.6 (1.2)	0.321
**Respiratory rate resp/min, median (interquartile range)**	16.4 (3.3)	15.7 (2.9)	0.289
**PCR Ct gen N, median (interquartile range)**	20.5 (19.2 – 24.6)	21.5 (18.7 – 24.6)	0.945
**PCR Ct gen E, median (interquartile range)**	18.8 – (16.9 – 22.4)	19.3 (17.1 – 22.3)	0.747
**Reinfection log delta-Ct, mean (SD)**	10^2.6^ (10^6.1^)	10^3.4^ (10^6.4^)	0,559

SD, Standard deviation; PDT, Photodynamic Treatment. Bold values = p<0.05.

**Figure 2:**
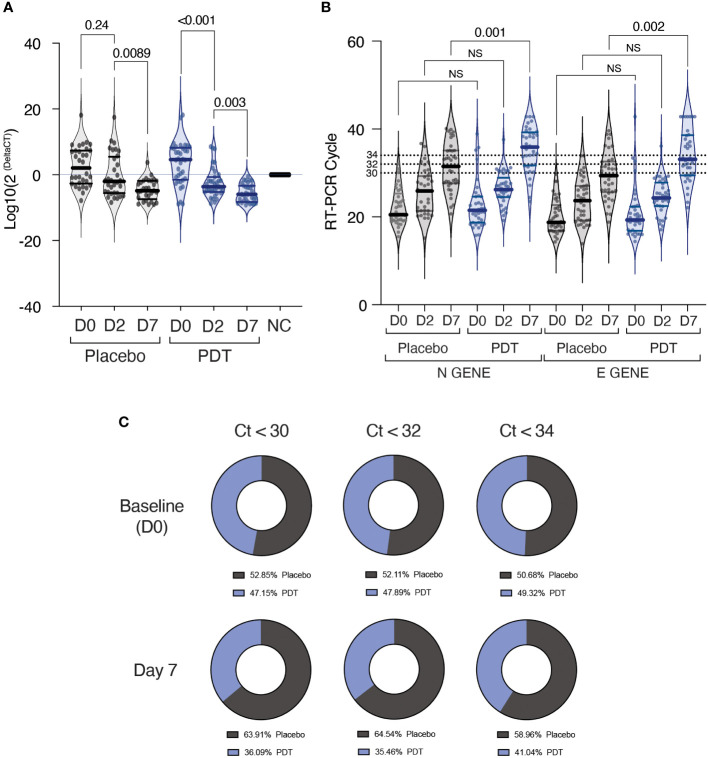
Results of infectivity assays and RT-PCR on Nasopharyngeal Swabs. **(A)** Infectivity assays. Subjects recruited in the study underwent nasopharyngeal swabs at baseline (Day 0), at day 2 and at day 7. The sample was collected in Viral transport medium and was subsequently used for the analysis of infectivity on VeroE6 cells. Seventy-two hours after infection, the supernatant was tested for the presence and quantity of SARS-CoV-2 using RT-PCR for Orf1b gene and human beta actin. Data is expressed in log10 of the delta between beta actin and Orf1b gene fluorescence threshold cycle. A Wilcoxon test for paired data was used for comparing baseline to D2 and D2 to D7. **(B)** RT-PCR of E and N genes of SARS-CoV-2 from nasopharyngeal swab samples immediately after collection. Data is expressed in fluorescence threshold cycle. A Wilcoxon test for paired data was used for comparing baseline to D2 and D2 to D7. **(C)** Percentage of “positive” and “negative” individuals in each of the two groups at baseline, D2 and D7, depending on which cycle threshold was chosen. NS, Non Significant; NC, Negative Control; PDT, Photodynamic Therapy.

Patients in the PDT arm showed a significant decrease in the *in vitro* infectivity measured by day 2 (Median of decrease: 61.75 (IQR: 0.14-1586) p<0.0001), while placebo patients did not (Median of decrease: 0.08 (IQR: -3.92-1150) p=0.24) **(** **Figure 2A**). Both arms demonstrated a significant decrease in infectivity by day 7. **(** **Figure 2A**). In the multi-adjusted linear regression model, PDT-treatment demonstrated a protective effect at 3 days with a mean β coefficient of -812 (95%CI -478660- -1.3, p<0.05) and a trend at 7 days with a mean β coefficient of -9.3 (95%CI -269.1- 3.1, p NS) (**Table 2**).

**Table 2:** Risk of infectivity capacity according to PCR test delta-Ct at days 2 and 7.

Risk of infectivity capacity according to PCR test delta-Ct 2 and 7.		
**Test**	**Placebo**	**PDT**
N	31	36
Day 2 PCR test delta-Ct, β coefficient (95%)*	1(ref.)	**-812 (95%CI -478660- -1.3)**
Day 7 PCR test delta-Ct, β coefficient (95%)*	1(ref.)	-9.3 (95%CI -269.1- 3.1)
Risk of having a transmission capacity at 7 days depending on the antigen test and different Cts cut-off.		
**Test**	**Placebo**	**PDT**
N	38	37
Antigen test positive, OR CI (95%)*	1(ref.)	0.26 (0,08-0.84)
PCR test Ct <30, OR CI (95%)*	1(ref.)	0.28 (0,09-0.84)
PCR test Ct <32, OR CI (95%)*	1(ref.)	0.16 (0.05-0.52)
PCR test Ct <34, OR CI (95%)*	1(ref.)	0.15 (0.04-0.58)

*Adjusted by sex, age, number of symptoms, COVID vaccine, Booster dose of COVID vaccine, and reinfection of SARS-CoV-2.

PDT, Photodynamic Therapy. Bold values = p<0.05.

During the study, both groups presented increased PCR cycle counts, although PDT-treated patients showed a significant difference in both E and N genes at day 7. **(** **Figure 2B**). When analysing the probability of being PCR positive at day 7, nasal PDT showed a protection against PCR positivity with Odds Ratio of 0.16 (95% CI: 0.05-0.52) and 0.15 (95% CI: 0.04-0.58), using PCR thresholds of 32 or 34 cycles respectively **(** **Table 2**; **Figure 2C** **)**.

**Figure 3:**
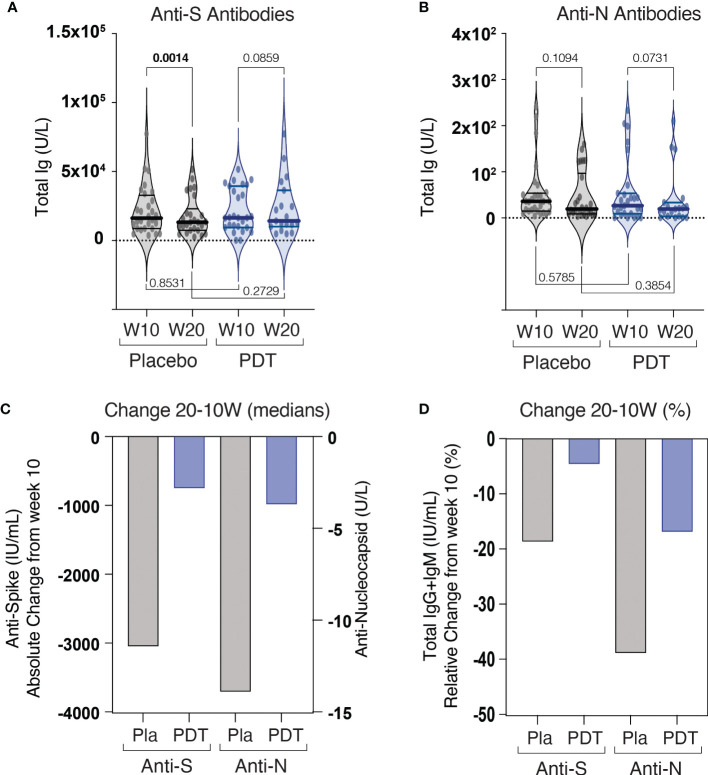
Results of humoral immunity assays. Patients were followed up after intranasal PDT treatment for 20 weeks. On week 10 and week 20, a sample of plasma and peripheral CD4 and CD8 T lymphocyte was collected **(A)** Detection of plasma total anti-Spike antibodies. **(B)** Detection of plasma total anti-Nucleocapsid antibodies. **(C)** Change of median levels at 20 weeks when compared to 10 weeks. **(D)** Percentage of change of median antibody levels at 20 weeks with respect to 10 weeks. Anti-S, Anti-Spike, Anti N, Anti Nucleocapsid, W10, 10^th^ week after End of treatment, W20, 20^th^ week after End of treatment, PDT, Pthotodynamic Treatment.

**Figure 4:**
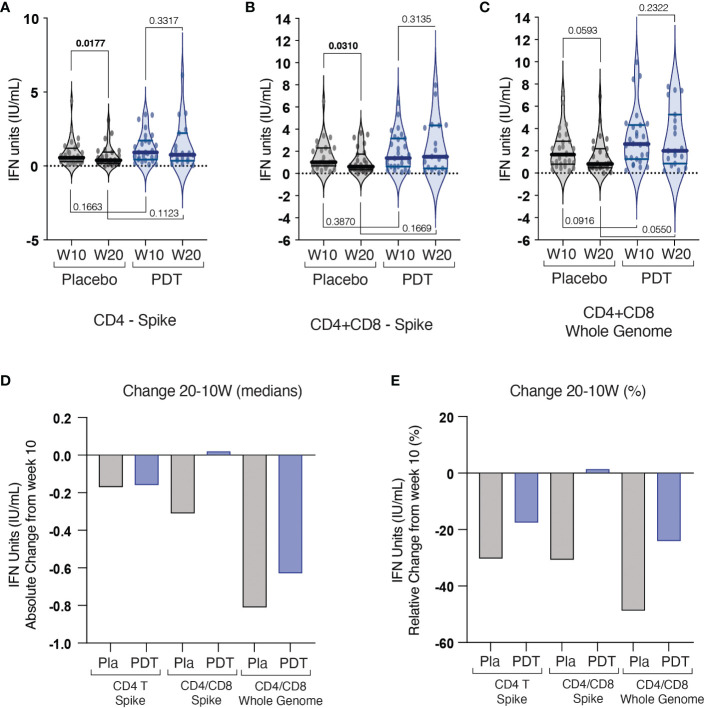
Results of cellular immunity assays. Quantiferon assay was used to analyse the specific T-cell responses against specific purified peptides from the spike antigen (S1, S2, RBD subdomains) either using isolated CD4 **(A)** or a combination of CD4/CD8 **(B)** T cells from placebo and PDT-treated subjects, or against additional specific peptides from the full genome of SARS-CoV-2 (S, N and M domains) testing both CD4 and CD8 responses **(C)**. **(D)** Change of median values of IFN units at 20 weeks when compared to 10 weeks for the three assays. **(E)** Percentage of change of median IFN units at 20 weeks when compared to 10 weeks. Pla, Placebo; PDT, Photodynamic Therapy; W, Week; IFN, Interferon gamma.

Antibody quantification at 10 and 20 weeks showed no differences in the production of anti-spike (**Figure 3A**) and anti-nucleocapsid (**Figure 3B**) antibodies, between control and PDT arms (**Supplementary Table 1**). There was a significant decay in Anti-Spike antibodies at 20 weeks in the placebo group (p=0.001), but not in the PDT group (p= 0. 0859) (**Figures 3A, C, D**). The measurement of interferon (IFN) synthesis by CD4 T cells after exposure to SARS-CoV-2 spike antigen revealed lower median IFN units in the placebo group when compared to PDT. (**Supplementary Table 2**; **Figure 4A**). CD4 and CD8 T-cell responses against the Spike protein were also trending higher in PDT-treated individuals with medians at 20 weeks almost double than placebo (p = 0.0971) (**Supplementary Table 2**; **Figure 4B**). When CD4 and CD8 cells were exposed to SARS-CoV-2, a trend for increased T-cell responses were observed in the PDT group when compared to placebo, at 10 and 20 weeks (p = 0.0916 and p = 0.0550) (**Supplementary Table 2**; **Figure 4C**). The decay of specific T-cell immunity against SARS-CoV-2 from week 10 to 20 tended to be of higher significance in the control group (p = 0.0593), than in the PDT group (p = 0.232) (**Figures 4C–E**).

COVID-19 RNA sequencing was successful in 72 patients (96%). The results indicate that 97.2% of patients were infected with Omicron 21K variant (B.1.1.529 or BA.1) one with Omicron 21L (BA.2) and one with Delta 21J (**Supplementary Figure 1**)

When analysing the progression of symptoms at day 3 and 7, a significant decrease in chest tightness and headache was observed in the PDT-treated group (**Table 3**).

**Table 3:** COVID19 symptoms detected during the clinical trial.

Signs/symptoms	Day 0			Day 2			Day 7			Day 14		
	Placebo	PDT	p	Placebo	PDT	p	Placebo	PDT	p	Placebo	PDT	p
**Sore throat (%)**	65.8	54.1	0.300	31.6	21.6	0.330	10.5	13.5	0.691	7,9	0	0,081
**Chills/dysentery (%)**	44.7	35.1	0.396	10.5	5.4	0.414	2.6	1.3	0.321	0	0	
**New or worsening cough (%)**	68.4	70.3	0.862	60.5	51.4	0.424	39.5	35.1	0.698	18,4	18,9	0,956
**Respiratory distress (%)**	5.3	10.8	0.376	10.5	8.1	0.719	10.5	8.1	0.719	7,9	2,7	0,317
**Chest tightness (%)**	13.2	5.4	0.249	**15.8**	**2.7**	**0.05**	13.2	5.4	0.249	5,3	2,7	0,572
**Temperature over 38° Celsius (%)**	15.8	5.4	0.145	5.3	0	0.157	0	0		0	0	
**Fatigue (%)**	50	45.5	0.411	34.2	24.3	0.347	18.4	21.6	0.729	23,7	16,2	0,419
**Muscle pain (%)**	47.4	43.2	0.720	26.32	16.22	0.286	5.3	5.4	0.978	5,3	2,7	0,572
**Loss of smell (%)**	0	2.7	0.308	5.3	5.4	0.978	2.6	5.4	0.540	2,6	0	0,321
**Loss of taste (%)**	2.6	5.4	0.540	2.63	10.81	0.156	2.6	10.8	0.156	5,3	0	0,157
**Headache (%)**	71.1	54.1	0.128	**39.5**	**16.2**	**0.025**	18.4	16.2	0.801	7,9	5,4	0,666
**Gastrointestinal symptoms (%)**	34.2	18.9	0.134	15.8	8.1	0.306	10.5	8.1	0.719	2,63	5,4	0,54
**Difficulty sleeping (%)**	29	21.6	0.466	10.5	16.2	0.469	2.6	5.4	0.540	2,6	2,7	0,985
**General malaise (%)**	65.8	51.4	0.204	31.6	19	0.208	7.9	16.2	0.268	2,6	5,4	0,540
**Nasal congestion (%)**	84.2	86.5	0.781	68.4	70.3	0.862	57.9	48.7	0.422	**36,8**	**16,2**	**0,043**

PDT, Photodynamic Therapy. Bold values = p<0.05..

Regarding intervention safety, 32 patients (5 in the control group and 27 in the intervention group) reported a total of 53 mild adverse events. One patient in the intervention group dropped out due to local itching after first PDT (**Supplementary Figure 2**).

## Discussion

A 3-day treatment with intranasal PDT can reduce SARS-CoV-2 infectivity and induce specific T-cell responses in COVID-19 patients. When applied in early stages of SARS-CoV-2 infection, local decolonization of nasal passages could be clinically relevant for the inhibition of viral spread both from person to person and from upper to lower respiratory tract, in clinical and non-clinical settings. PDT has been proposed as a valid approach to inactivate SARS-CoV-2 (Almeida et al., 2020; Dias et al., 2020; Sabino et al., 2020; Ziganshyna et al., 2022), and the ex-vivo efficacy of PDT against SARS-CoV-2 virion has been demonstrated (Arentz and von der Heide, 2022). This study is the first prospective randomized trial exploring the safety and efficacy of PDT nasal decolonization in SARS-CoV-2 infected individuals.

The primary endpoint of the trial (infectivity reduction) was achieved. We demonstrated a reduction of infectivity in mildly symptomatic, otherwise-healthy individuals with high viral load (Ct<27), when applied during the first days of the replication phase in the nasal mucosa. The treatment consisted of three consecutive day, 12 min-applications of methylene blue-based PDT. Because of the absence of prior literature in intranasal PDT treatment of SARS-CoV-2, we decided to adopt a repeated application protocol rather than the single- or double-applications tested *in vitro*. This was supported by the low toxicity of the technique, the expected high viral replication rate, the intracellular life of viruses and the interference of mucous layers, especially in patients with nasal congestion. Whether an intensified protocol could demonstrate further-enhanced efficacy while maintaining acceptable local toxicity, should be explored in future studies. Interestingly, although the treatment with PDT was applied only to the nasal passages, it also reduced SARS-CoV-2 colonization of pharyngeal region. This result strengthens the hypothesis of the importance of early nasal decolonization in a disease that could develop to a systemic stage in few days.

The effects of PDT were observed as soon as 3 days after treatment using an *in vitro* infectivity assay with NP swabs. PCR thresholds were also higher in the treated group at day 7, a further sign of the durable effect of nasal decolonization. Consequently, PDT treatment demonstrated an important protective effect against PCR positivity. There were no serious adverse effects reported during treatment.

We observed a sharp decrease in viral load in our entire population (placebo and PDT) between day 3 and 7, which presumably masked the true magnitude of the PDT. This was probably related to the fact that vaccination is associated with faster viral RNA decline (Kissler et al., 2021; Chia et al., 2022) and that almost all patients were infected by Omicron variant (21K, BA.1) (97.2%) with a favourable biology (Bouzid et al., 2022). Despite these two masking effects, patients in the PDT arm demonstrated significantly higher elimination rates of viral particles.

The titer of anti-S and anti-N antibodies at 10 and 20 weeks after treatment was not different between the control and the intervention group. In both groups the amount of anti-S and anti-N antibody decreased at 20 weeks, revealing the decline of humoral response already described in other studies of SARS-CoV-2 infection (Fernandez-Ciriza et al., 2022). However, at week 20, the decrease in anti-S antibody was significant only in the control group. This could suggest a deceleration of the natural decline of specific B-cell responses by PDT treatment, although this concept should be confirmed in future studies with larger cohorts. Although the median decrease of anti-N antibody was also higher in the placebo group than in the PDT group, this did not reach statistical significance due to the large dispersion of the data in both groups.

Similar to what we found in the humoral response, no significant differences were seen in the specific T-cell immunity against SARS-CoV-2 between control and intervention groups. Paralleling the humoral response, subjects in both groups experienced a reduction of the specific T cell responses between the week 10 and 20. Interestingly, the decline was significant only in placebo-treated patients for both CD4 and CD4+CD8 anti-S T-Cell responses, but not in PDT-treated subjects (**Figures 4A, B**; **Supplementary Table 2**). Regarding the CD4+CD8 anti-whole SARS-CoV-2 genome the median interferon produced by T Cells was superior in PDT-treated subjects, with trending p values bot at 10 weeks and at 20 weeks (**Figure 4C** and **Supplementary Table 2** **)**. The relevance of the effect of nasal PDT treatment on preventing the natural decline and/or inducing T cell immunity against SARS-CoV-2, has not been described so far. One hypothesis is that the destruction of viral particles in the nasal mucosa, could enhance the uptake, processing, or presentation of viral peptides by Antigen Presenting Cells (APC). In the field of cancer vaccines, the use of PDT enhanced adjuvant-free cross-priming of APC to CD8 T lymphocytes (Mroz et al., 2010). In a β-galactosidase-bearing colon adenocarcinoma model, the use of vascular PDT induced the remission of tumors. T lymphocytes isolated from cured mice recognized the β-galactosidase-derived epitopes and kill only cancer cells expressing β-galactosidase. The anti-tumoral response to PDT treatment was able to eliminate distant untreated antigen-expressing lesions (Huang et al., 2012). These data suggest that PDT could also induce APC uptake of SARS-CoV-2 antigens in the nasal mucosa of infected patients. Future preclinical studies should be directed to test this hypothesis. If confirmed, this effect of PDT may be of great relevance in reducing transmission of future viral variants, where immune escape is otherwise almost certain.

Other studies with different local treatments have been performed. A double-blind, randomized, placebo-controlled trial in patients with mildly symptomatic SARS-CoV-2 infections (n=306) of treatment with nitric oxide nasal spray (NONS) was evaluated. NONS was self-administered six times daily for 7 days (Schikora et al., 2020). This work had several similarities with our study: both evaluated inactivation of nasal viral load through local treatments to shorten infectivity, and both utilized a similar population of non-severe SARS-CoV-2 patients. However, the patients in the NONS trial were only tested by RT-PCR while our study used both RT-PCR and infectivity assay, increasing both sensitivity and relevance to downstream transmission (Jamal et al., 2021; Berenger et al., 2022). Second, the NONS study included ~ 60% unvaccinated patients and did not include follow-up, while our study included follow-up visits to demonstrate absence of viral replication rebound. Finally, the impact of the NONS treatment on anti-SARS-CoV-2 immunity is unknown, whereas this was convincingly demonstrated in our PDT study.

The present study had several limitations, the power of the study was planned for the infectivity assay, but full recruitment was unable to be completed due to the decrease in SARS-CoV-2 cases in February 2022 in Spain. The study was not powered to detect differences in the IFN assays, but based on the differences observed between groups, a higher sample size would probably reveal consistently significant results. Finally, the present study only incorporated individuals infected with the Omicron strain. In future studies, to better characterize the immunological effect of PDT in clinical setting, flow cytometry studies could be used to describe the activation of innate immune cells (including NK and Dendritic Cells) and the specific phenotype of CD4 T lymphocytes (naïve, regulatory or memory T cells), before and after PDT treatment. In addition, circulating cytokines and chemokines, especially at short times, could help in defining the immune landscape of these patients, and whether the treatment with PDT can alter it.

Strengths of the study included the high retention rate, the follow-up period and the infectivity assays along with humoral and cellular immunity evaluations up to 20 weeks after infection, all of which help to better elucidate the effect of nasal PDT on SARS-CoV-2 colonization and immunization. The impact of PDT in different settings such as ENT or dentistry clinics, Intensive Care Units, first responder settings, or GI laboratories where contact with potentially infectious patients could jeopardize efficiency of diagnostic and therapeutic procedures warrants further investigation. Development of new affordable and wearable nasal illuminators might provide an alternative, low-cost, widely accessible, respiratory virus treatment during future pandemics.

## Conclusion

PDT treatment was well tolerated, and no serious adverse effects were detected. Nasal PDT enhances SARS-CoV-2 viral clearance in mild COVID-19 patients, even in vaccinated individuals, rapidly decreasing infectivity. Nasal PDT decelerates the decline of SARS-CoV-2 specific T-cell immune responses in vaccinated individuals infected with Omicron variant.

## Data availability statement

The raw data supporting the conclusions of this article will be made available by the authors, without undue reservation.

## Ethics statement

The studies involving human participants were reviewed and approved by the Institutional Review Board of the University of Navarra (registry number: EC_2021/1). The patients/participants provided their written informed consent to participate in this study.

## Author contributions

JA, FC-T and AF-M conceived the study, designed the protocol, submitted the requested changes the Institutional Board Review for its approval, organized the field work, discussed the results, and wrote the manuscript. JK helped on the trial design. AF-M recruited and randomized the patients. JZ, MP-S, LM-N, NM-R, MP, MM-F treated and followed patients. MR organized and performed the RT-PCR studies. GR organized and performed the immunological assays. JP and FC-T reviewed the microbiological and immunological data. JQ, CS, GG-A, LM-G, and JP reviewed the manuscript. CS, SM, CO, GC, JR. and GG-A coordinated and performed the *in vitro* infectivity assays. FM, JZaz and MPoz prepared the libraries, did the sequencing and performed variant analysis. All authors contributed to the article and approved the submitted version.

## Supplementary material

Supplementary Table 1 Antibody assays.

Supplementary Table 2 T Cell immunity assays.

Supplementary Figure 1 Phylogeny of viral species from patients’ samples.

Supplementary Figure 2 Local ENT adverse effects during the clinical trial.
