# Supplementary material for: Photodynamic nasal SARS-CoV-2 decolonization shortens infectivity and influences specific T-Cell responses
Source: Front Cell Infect Microbiol. 2023 Jan 25;13:1110467. doi: 10.3389/fcimb.2023.1110467 (PMC9905247; doi:10.3389/fcimb.2023.1110467)
Supplement: Supplementary Table 1 — Antibody assays. [file Table_1.docx]

**Supplementary table 1. Antibody Assays.**

|  | **At 10 weeks**  **(median, IQR)** | | **At 20 weeks**  **(median, IQR)** | | Delta (20w-10w) | | **U-Mann Whitney** | | **Wilcoxon** | |
| --- | --- | --- | --- | --- | --- | --- | --- | --- | --- | --- |
| **Test** | Placebo | PDT | Placebo | PDT | Placebo | PDT | P Value  Placebo vs PDT 10w | P Value  Placebo vs PDT 20w | P Value  Placebo  10w vs 20w | P Value  PDT  10w vs 20w |
| **No.** | 28 | 28 | 25 | 20 |  |  |  |  |  |  |
| **Anti-spike Antibody (titer)** | 16278 (8703 – 31416.5) | 16459 (9422 – 39436) | 13229 (7469 -19782) | 15707 (10744 – 30704) | -3049 (-18.7%) | -752 (-4.6%) | 0.8531 | 0.2729 | **0.0014** | 0.0859 |
| **Anti-nucleocapsid Antibody (Index)** | 35.7 (14.7 – 52.2) | 26.6 (9.1 – 53.5) | 21.8 (8.9 - 88.4) | 18.1 (3.7 - 28.9) | -13.9 (-38.9%) | -3.7 (-16.9%) | 0.5785 | 0.3854 | 0.1094 | 0.0731 |

PDT: Photodynamic Therapy

**Supplementary table 2. T Cell Immunity Assays.**

|  | **At 10 weeks**  **(median, IQR)** | | **At 20 weeks**  **(median, IQR)** | | Delta (20w-10w) | | **U-Mann Whitney** | | **Wilcoxon** | |
| --- | --- | --- | --- | --- | --- | --- | --- | --- | --- | --- |
| **Test** | Placebo | PDT | Placebo | PDT | Placebo | PDT | P Value  Placebo vs PDT 10w | P Value  Placebo vs PDT 20w | P Value  Placebo  10w vs 20w | P Value  PDT  10w vs 20w |
| **No.** | 28 | 27 | 25 | 20 |  |  |  |  |  |  |
| **CD4 T cell to Spike (IFN units/mL)** | 0.56 (0.29 – 1.2) | 0.91 (0.42 – 1.71) | 0.39 (0.19 – 0.92) | 0.75 (0.34 – 1.78) | -0.17 (-30.3%) | -0.16 (-17.6%) | 0.1663 | 0.1123 | **0.0177** | 0.3317 |
| **CD4/CD8 T cell to Spike (IFN units/mL)** | 1.01 (0.69 – 2.31) | 1.39 (0.63 – 3.16) | 0.7 (0.39 – 1.7) | 1.41 (0.4 – 4.23) | -0.31 (-30.7%) | +0.02 (+1.4%) | 0.3870 | 0.1669 | **0.0310** | 0.3135 |
| **CD4/CD8 T cell to Whole Genome** | 1.66 (0.8 – 2.87) | 2.61 (1.26 – 4.31) | 0.85 (0.49 – 2.18) | 1.98 (0.76 – 5) | -0.81 (-48.8%) | -0.63 (-24.1%) | 0.0916 | 0.0550 | 0.0593 | 0.2322 |

PDT: Photodynamic Therapy

**Supplementary figure 1. Phylogeny of Viral Species from patients’ samples.**

**Supplementary figure 2. Local ENT adverse effects during the clinical trial.**
