# Supplementary figures and images for: Photodynamic nasal SARS-CoV-2 decolonization shortens infectivity and influences specific T-Cell responses
Source: Front Cell Infect Microbiol. 2023 Jan 25;13:1110467. doi: 10.3389/fcimb.2023.1110467 (PMC9905247; doi:10.3389/fcimb.2023.1110467)

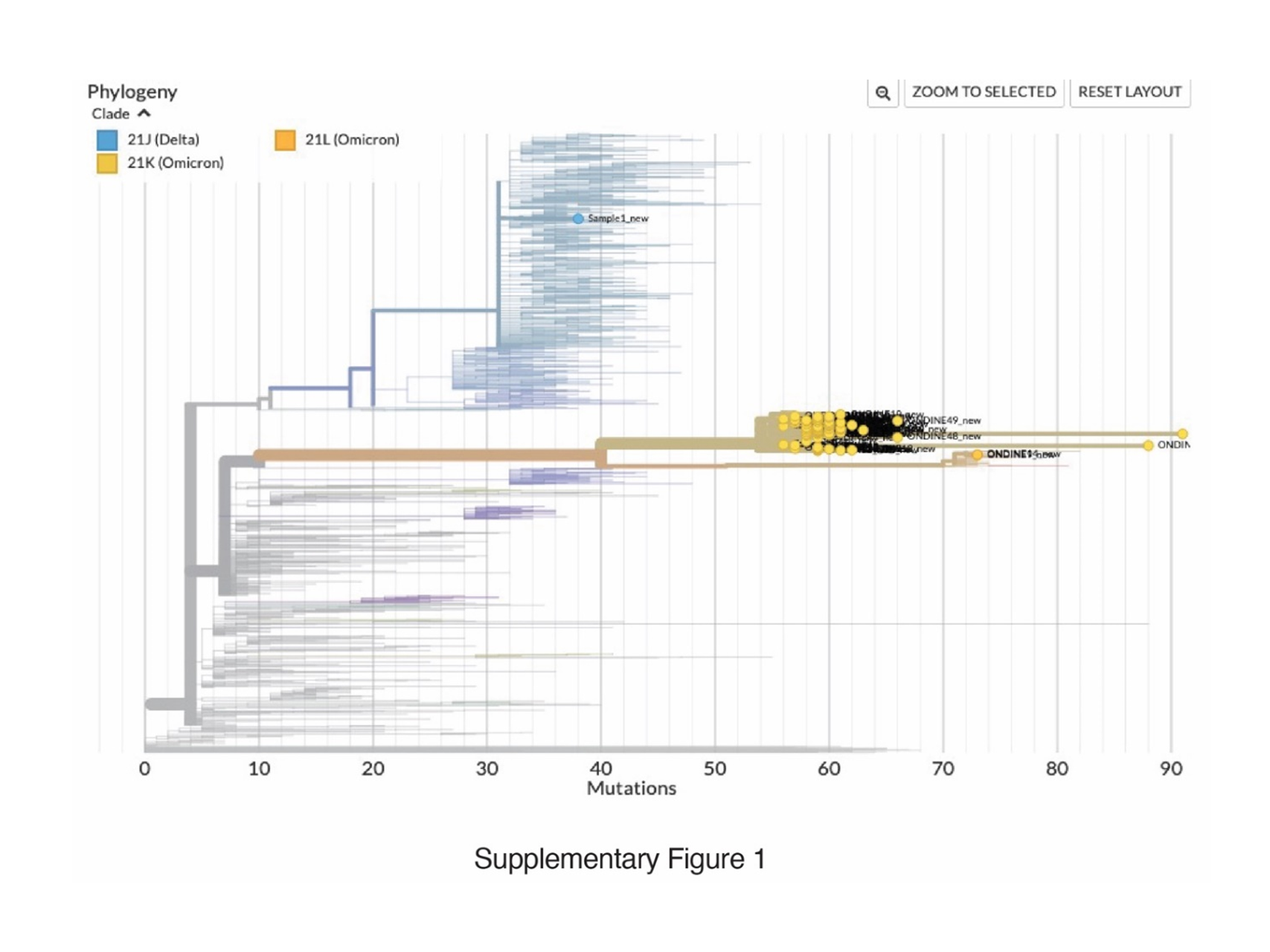

Supplement: Supplementary Figure 1 — Phylogeny of viral species from patients’ samples. [file Image_1.tif]

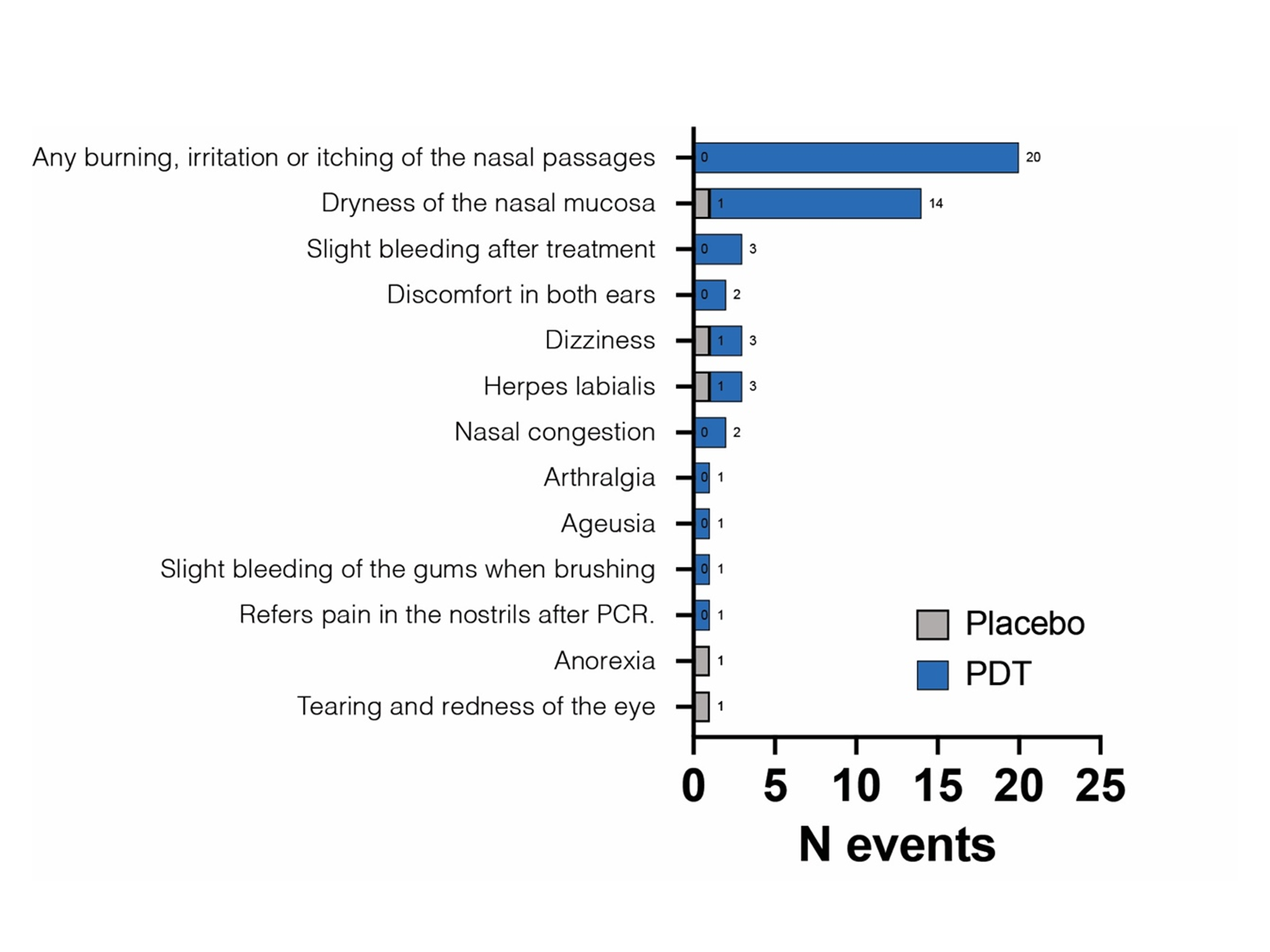

Supplement: Supplementary Figure 2 — Local ENT adverse effects during the clinical trial. [file Image_2.tif]
